# A rare congenital triad: ebsteinoid tricuspid valve, giant right atrial aneurysm, and atrial septal defect

**DOI:** 10.1186/s43044-026-00787-8

**Published:** 2026-09-27

**Authors:** Uma Devi Karuru, Sunitha Arumulla, Neusha Doddi, Rama Kumari Nuthalapati

**Affiliations:** Nizam’s Institute of Medical Sciences, Hyderabad, India

**Keywords:** Ebsteinoid anomaly, Ebstein anomaly, Right atrial aneurysm, Tricuspid valve repair, Atrial septal defect

## Abstract

**Background:**

Ebstein anomaly is a rare congenital malformation of the tricuspid valve, accounting for less than 1% of all congenital heart diseases. Ebsteinoid anomaly is an uncommon morphological variant characterized by abnormal tricuspid valve leaflet morphology without the classical apical displacement of the septal leaflet seen in Ebstein anomaly. The coexistence of an Ebsteinoid anomaly, giant right atrial aneurysm (RAA), and atrial septal defect (ASD) is exceptionally rare.

**Case presentation:**

A 15-year-old girl presented with progressive exertional dyspnoea (New York Heart Association [NYHA] class II) and intermittent palpitations. Chest radiography demonstrated marked cardiomegaly. Transthoracic echocardiography revealed a giant right atrium, severe low-pressure tricuspid regurgitation, an ostium secundum ASD, and an Ebsteinoid tricuspid valve with an elongated anterior leaflet and tethered septal leaflet, without apical displacement or right ventricular atrialization. Four-dimensional echocardiography and cardiac computed tomography confirmed the anatomical findings. Cardiac catheterization demonstrated pulmonary artery pressures approximately one-third of systemic pressure, confirming operability. The patient underwent successful tricuspid valve repair, ASD closure, and right atrial reduction atrioplasty. At one-year follow-up, she remained asymptomatic (NYHA class I) with preserved right ventricular function and only trivial residual tricuspid regurgitation.

**Conclusion:**

This case describes an exceptionally rare combination of Ebsteinoid tricuspid valve anomaly, giant right atrial aneurysm, and an ostium secundum ASD. Multimodality imaging was essential for accurate anatomical delineation and surgical planning. Early surgical correction resulted in excellent clinical and echocardiographic outcomes, underscoring the importance of timely intervention in complex congenital tricuspid valve anomalies.

**Supplementary Information:**

The online version contains supplementary material available at https://doi.org/10.1186/s43044-026-00787-8.

## Introduction

 Ebstein anomaly is an uncommon congenital malformation caused by incomplete delamination of the septal and posterior tricuspid valve leaflets, leading to tricuspid regurgitation, atrialization of the right ventricle, and progressive right-sided chamber enlargement [1–3]. Associated atrial septal defect (ASD) or patent foramen ovale occurs in approximately 50–80% of patients and contributes to chronic right-sided volume overload and arrhythmias [2, 4].

The term Ebsteinoid anomaly has been used to describe congenital tricuspid valve abnormalities resembling Ebstein anomaly but lacking the classical apical displacement of the septal leaflet. Because this entity is rarely reported, its natural history and optimal surgical management remain poorly defined [5, 6].

Massive right atrial enlargement secondary to severe tricuspid regurgitation is common; however, development of a true giant right atrial aneurysm is exceedingly uncommon [5, 6]. Such patients are at increased risk of atrial arrhythmias, thromboembolic events, progressive right ventricular dysfunction, and heart failure. Modern multimodality imaging including two-dimensional, three-dimensional and four-dimensional echocardiography, cardiac computed tomography (CT), and cardiac magnetic resonance imaging (MRI) plays a pivotal role in defining anatomy and planning surgery [3, 7].

We describe a rare case of a giant right atrial aneurysm secondary to an Ebsteinoid tricuspid valve anomaly associated with a secundum ASD in an adolescent successfully treated with tricuspid valve repair, ASD closure, and right atrial reduction atrioplasty.

## Case presentation

A 15-year-old girl presented with a five-year history of progressively worsening exertional dyspnoea and intermittent palpitations. Her functional status had gradually deteriorated from New York Heart Association (NYHA) class I to class II. There was no history of cyanosis, syncope, rheumatic fever, recurrent respiratory tract infections, or previous cardiac intervention.

On physical examination, a hyperdynamic right ventricular impulse was noted. Cardiac auscultation revealed a grade III/VI pansystolic murmur at the left lower sternal border that increased with inspiration, consistent with severe tricuspid regurgitation. There was no peripheral oedema, cyanosis, jugular venous distension, or hepatomegaly.

**Fig. 1:**
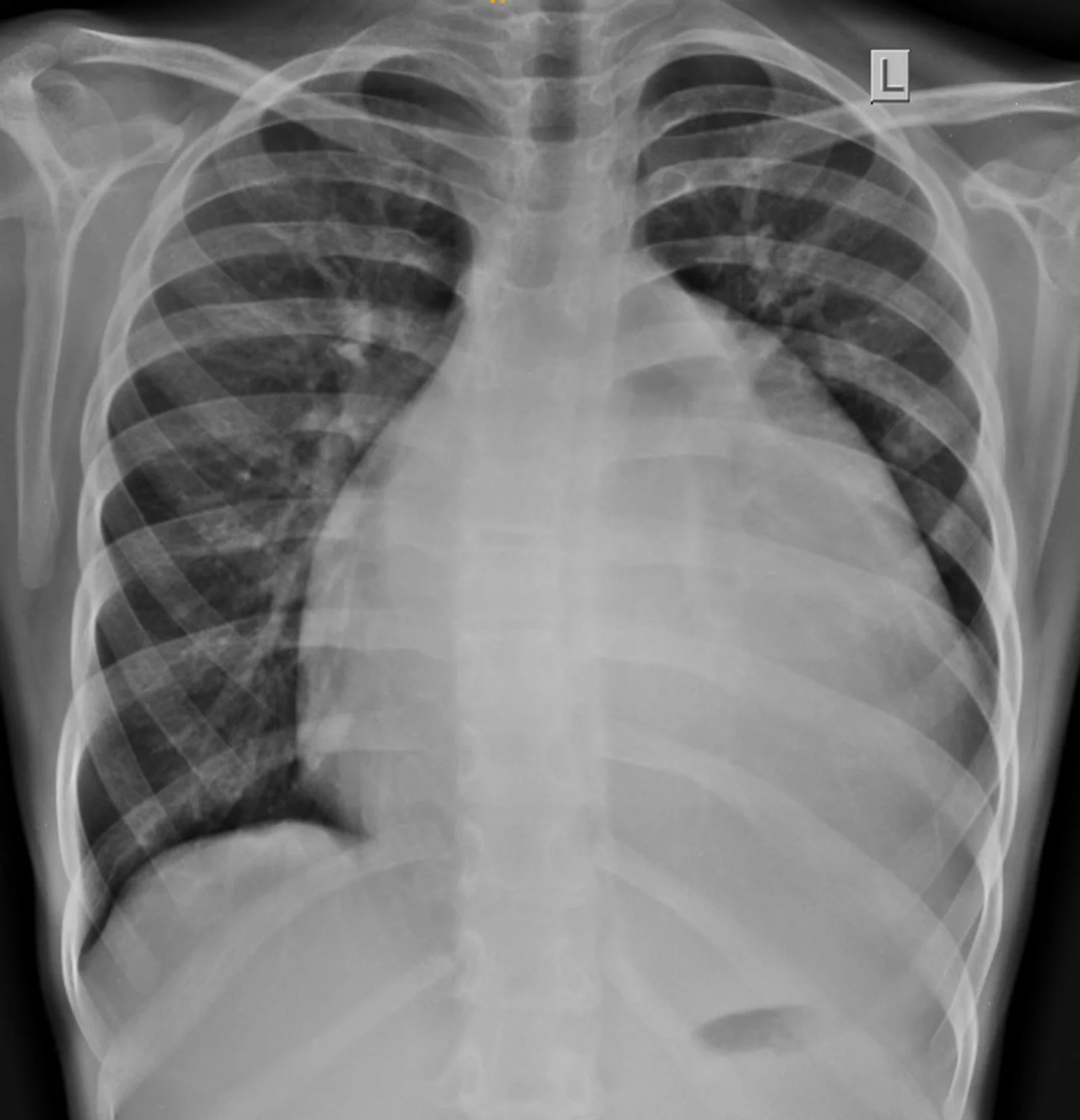
Chest radiograph (posteroanterior view) demonstrating marked cardiomegaly with elevated right ventricular apex and a cardiothoracic ratio of 75%. Prominent dilation of the right cardiac border indicates severe right atrial enlargement

Electrocardiography demonstrated sinus rhythm with right atrial enlargement, right-axis deviation, and incomplete right bundle branch block. Chest radiography showed marked cardiomegaly with prominence of the right cardiac border, consistent with severe right atrial enlargement, and a cardiothoracic ratio of 75% (Fig. 1).

**Fig. 2:**
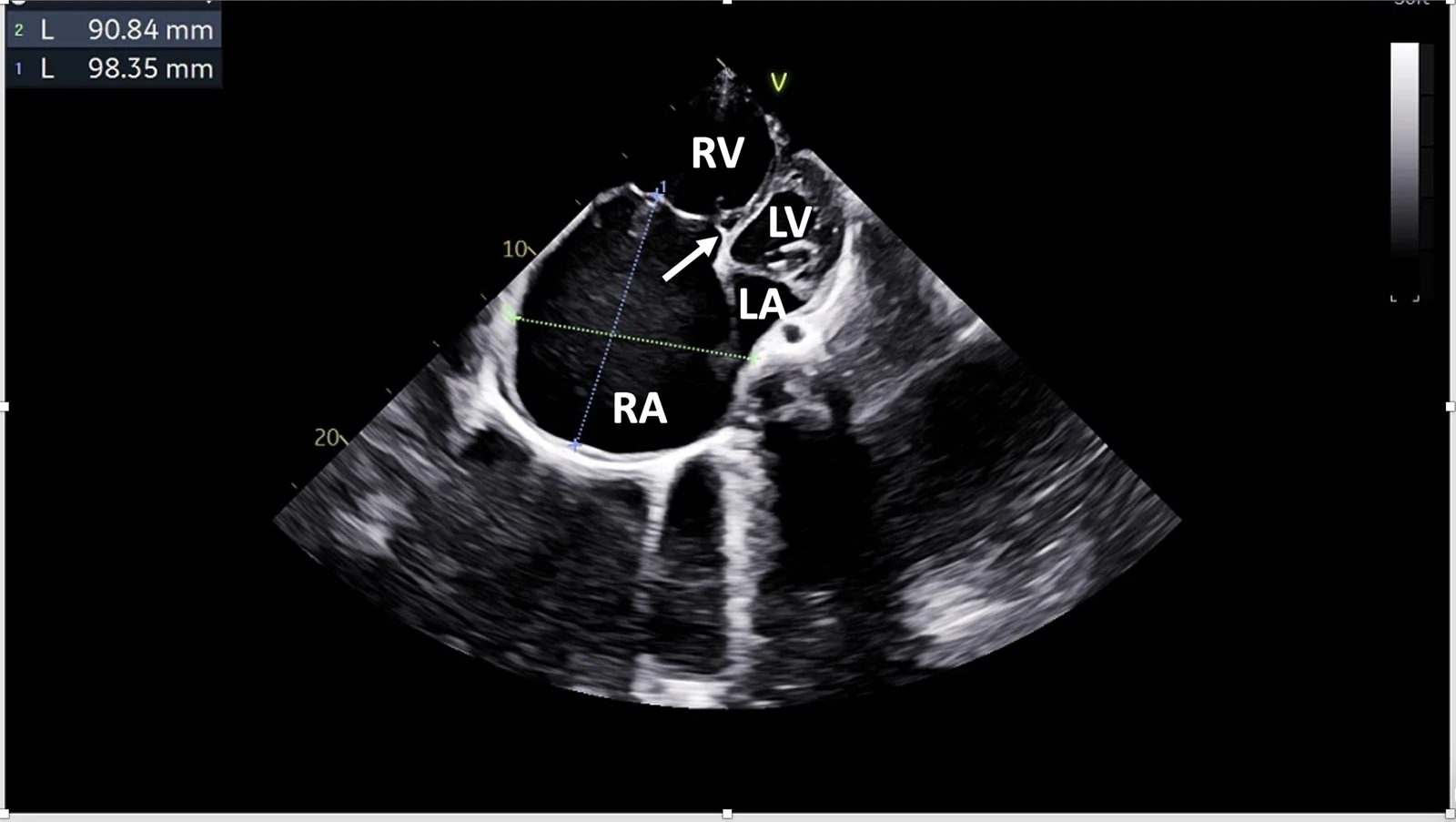
Transthoracic echocardiography (apical four-chamber view) showing a giant, aneurysmal right atrium measuring $\:90.8\mathrm{\:mm}\times\:98.4\mathrm{\:mm}$. The white arrow indicates the lack of typical apical displacement. *LA*,* left atrium; LV*,* left ventricle; RA*,* right atrium; RV*,* right ventricle*

**Fig. 3:**
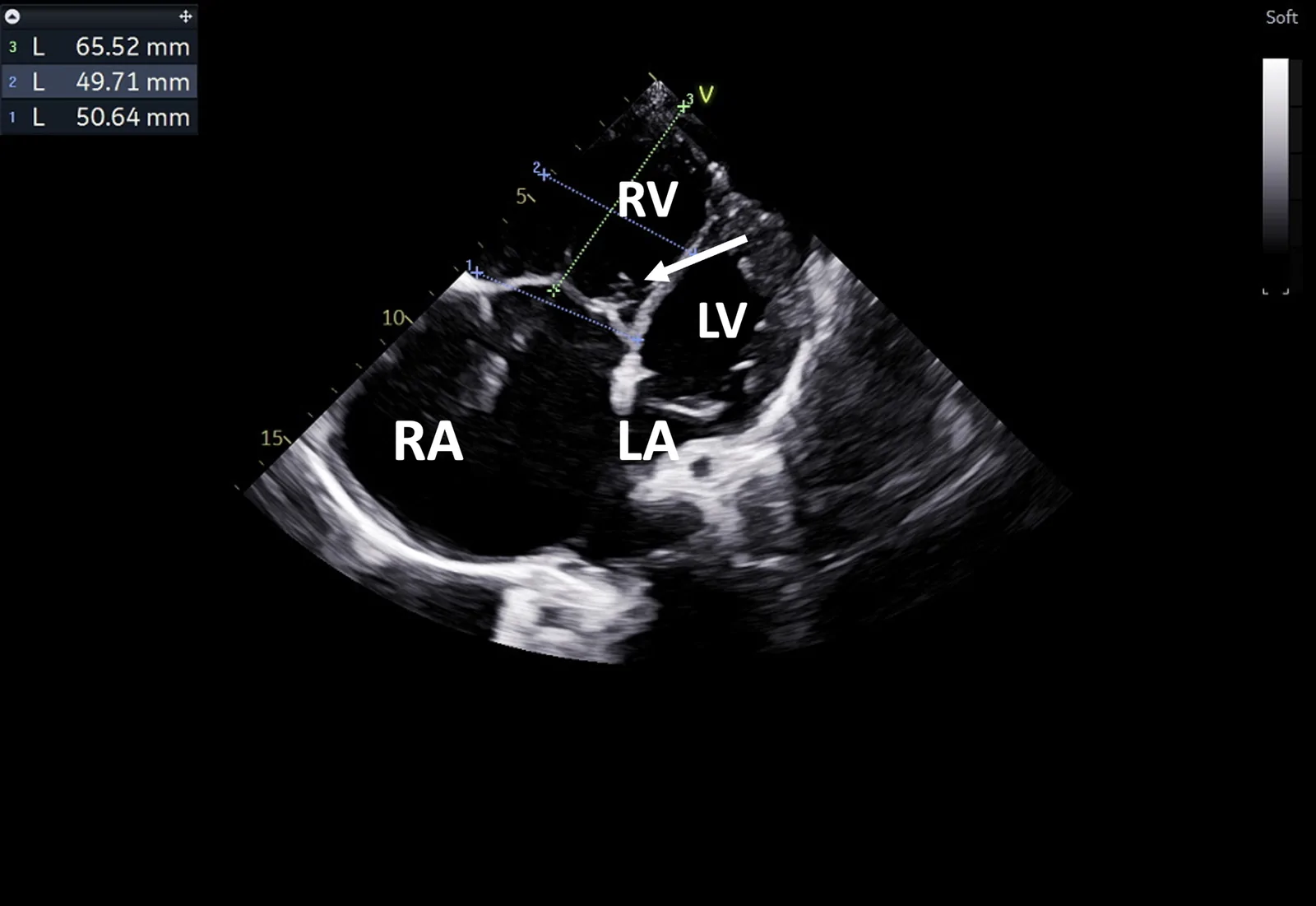
Transthoracic echocardiography (apical four-chamber view) detailing right ventricular dimensions ($\:49.7\mathrm{\:mm}\times\:50.6\mathrm{\:mm}\times\:65.5\mathrm{\:mm}$). The white arrow points to the tethered septal leaflet of the Ebstenoid tricuspid valve without typical apical displacement. *LA*,* left atrium; LV*,* left ventricle; RA*,* right atrium; RV*,* right ventricle*

**Fig. 4:**
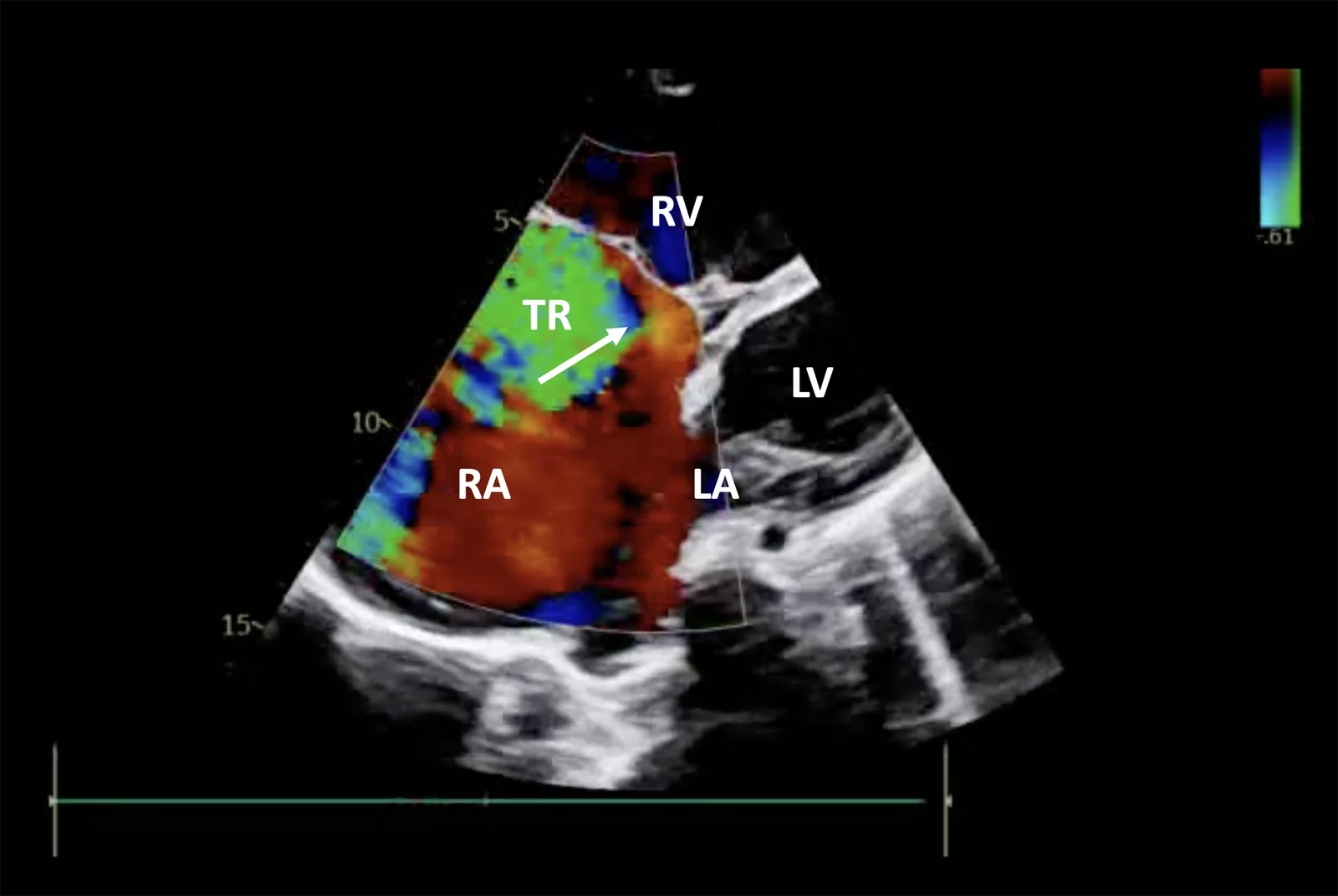
Transthoracic echocardiogram (apical four-chamber view) with color Doppler demonstrating severe, low-pressure tricuspid regurgitation (TR) filling the giant right atrium, with the regurgitant jet directed toward the interatrial septum (white arrow). *LA*,* left atrium; LV*,* left ventricle; RA*,* right atrium; RV*,* right ventricle; TR*,* tricuspid regurgitation*

**Fig. 5:**
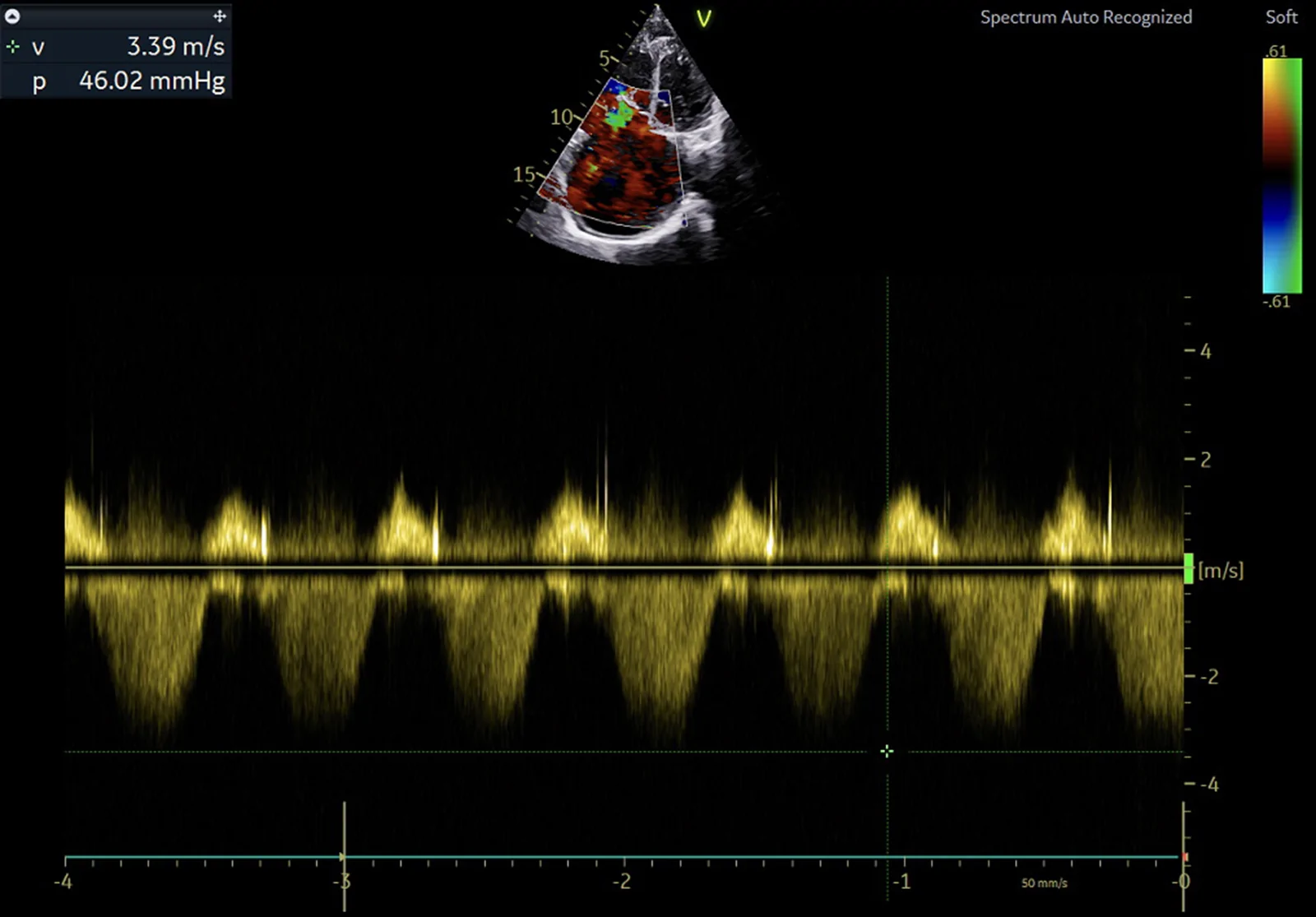
Continuous-wave Doppler interrogation of the tricuspid regurgitation jet yielding a peak systolic velocity of $\:3.39\mathrm{\:m/s}$ and an estimated right ventricular systolic pressure (RVSP) gradient of $\:46.0\mathrm{\:mmHg}$ (excluding right atrial pressure), indicative of moderate pulmonary hypertension

Transthoracic echocardiography revealed a massively dilated right atrium measuring **9.0 × 9.8 cm** and a dilated right ventricle measuring **4.9 × 5.0 × 6.5 cm**, with preserved right ventricular systolic function (Figs. 2 and 3). The tricuspid valve demonstrated an elongated anterior leaflet and a tethered septal leaflet without significant apical displacement, consistent with an Ebsteinoid anomaly. Color Doppler demonstrated severe, low-velocity, wide-jet tricuspid regurgitation (TR) with a broad vena contracta measuring 1.1 cm, directed eccentric-posteriorly into the aneurysmal atrium (Fig. 4, Videos 01). Continuous-wave Doppler across the TR jet demonstrated a peak velocity of 3.39 m/s, yielding a peak trans tricuspid systolic gradient of 46 mmHg (Fig. 5). In addition, a moderate ostium secundum ASD with left-to-right shunting was noted (Video 02).

**Fig. 6:**
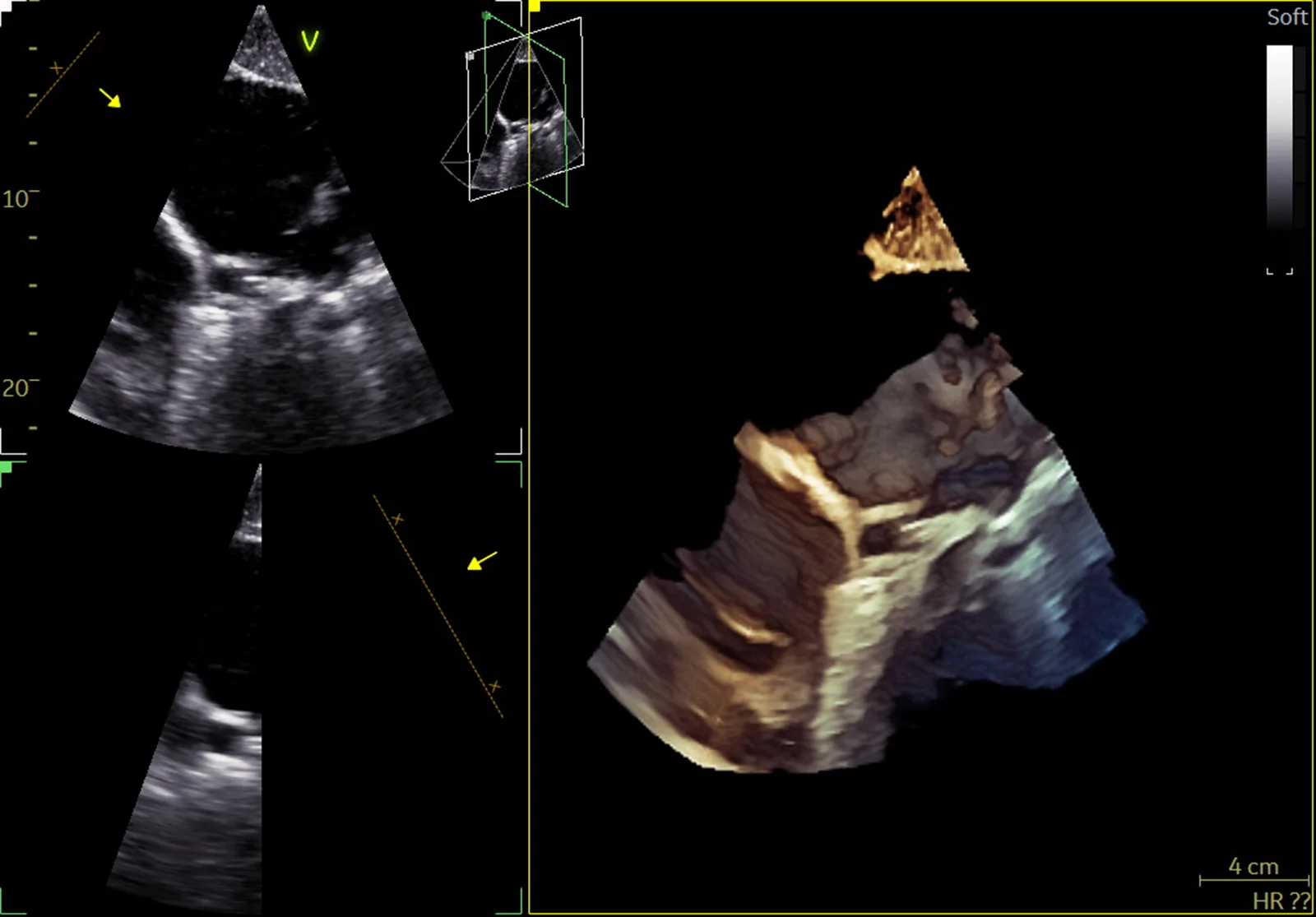
Four-dimensional transthoracic echocardiographic reconstruction viewed from the right atrial aspect, illustrating the massive aneurysmal cavity of the right atrium and abnormal tricuspid valve coaptation

**Fig. 7:**
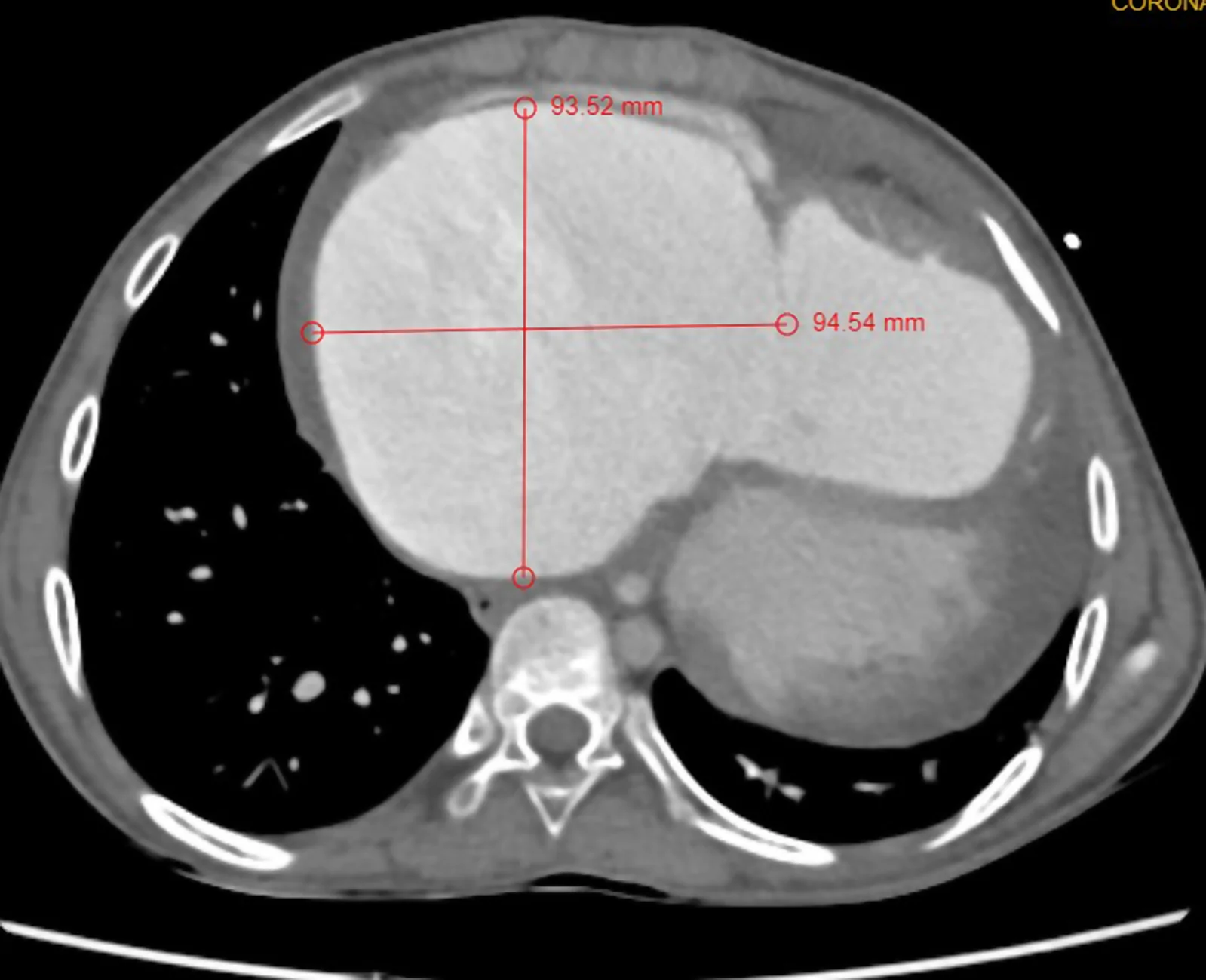
Contrast-enhanced axial cardiac computed tomography (CT) confirming a giant aneurysmal right atrium measuring $\:93.5\mathrm{\:mm}\times\:94.5\mathrm{\:mm}$ without evidence of intracavitary thrombus

Four-dimensional echocardiography provided detailed assessment of tricuspid valve morphology and leaflet coaptation and the aneurysmal atrial cavity (Fig. 6). Cardiac computed tomography confirmed a giant aneurysmal right atrium measuring **9.3 × 9.4 cm** (area **78 cm²**) without intracavitary thrombus and excluded associated congenital cardiovascular anomalies (Fig. 7). Diagnostic cardiac catheterization was undertaken to accurately evaluate hemodynamics and pulmonary vascular resistance. Catheterization revealed right atrial mean pressure: 8 mmHg, right ventricular pressure: 32/6 mmHg, pulmonary artery pressure: 28/12 mmHg (mean 18 mmHg), pulmonary capillary wedge pressure: 9 mmHg, pulmonary vascular resistance (PVR): 1.1 Wood units·m² and pulmonary-to-systemic flow ratio ($\:{Q}_{p}/{Q}_{s}$): 1.9:1.

Ventriculography and right heart angiography were intentionally deferred. High-resolution 2D/4D echocardiography and cardiac CT had already delineated the valve morphology, leaflet malcoaptation gap, and regurgitant severity, rendering invasive imaging redundant while avoiding excess contrast exposure and catheter-induced regurgitation artifacts across the tricuspid valve. Catheterization was reserved strictly for precise invasive hemodynamic profiling, which demonstrated favorable pulmonary vascular resistance and confirmed surgical operability.

Following multidisciplinary heart team discussion, the patient was scheduled for surgical correction because of progressive symptoms, severe tricuspid regurgitation, giant right atrial aneurysm, and the anticipated long-term risks of atrial arrhythmias, thromboembolism, and right ventricular dysfunction.

## Surgical management

The patient underwent elective surgery through a median sternotomy under cardiopulmonary bypass. Intraoperative inspection demonstrated an aneurysmal right atrium with paper-thin walls and severe annular dilatation. The tricuspid valve showed marked elongation of the anterior leaflet and tethering of the septal leaflet causing profound central and posterior malcoaptation, in the absence of downward apical leaflet displacement.

Native tricuspid valve repair was performed by mobilizing the anterior leaflet and systematically releasing restrictive tethering through selective resection of secondary, shortened chordae tendineae inserting into the ventricular septum. Annular dilatation was corrected and coaptation restored using a semi-rigid tricuspid annuloplasty ring (28 mm Carpentier-Edwards Physio tricuspid ring), anchored along the anterior and posterior annuluses while carefully sparing the conduction tissue across the triangle of Koch. To protect the conduction pathways during right atrial volume reduction, resection of the paper-thin, redundant aneurysmal wall was confined strictly to the anterolateral free wall. The superior margin of the resection was placed at least 1.5–2.0 cm lateral and inferior to the cavoatrial junction and crista terminalis to avoid injury to the sinoatrial (SA) node and its arterial blood supply. Inferomedially, the excision was carried out safely clear of the coronary sinus orifice and the triangle of Koch to safeguard the atrioventricular (AV) node. The remaining viable atrial tissue was re-approximated with a two-layer continuous polypropylene suture line, successfully reducing right atrial volume without precipitating nodal or junctional arrhythmias. Intraoperative transesophageal echocardiography confirmed a competent tricuspid valve with trivial residual regurgitation and no residual intracardiac shunt.

The postoperative course was uneventful. The patient remained in sinus rhythm without conduction disturbances. Serial echocardiography demonstrated trivial residual tricuspid regurgitation, preserved right ventricular function, and no residual atrial shunt. She was discharged in stable condition.

At one-year follow-up, the patient remained asymptomatic (NYHA class I). Echocardiography showed sustained reduction in right atrial size, stable tricuspid valve repair, preserved right ventricular function, and no recurrence of aneurysmal dilatation.

## Discussion

Ebstein anomaly encompasses a broad spectrum of congenital tricuspid valve abnormalities resulting from abnormal embryological delamination of the valve leaflets [1, 2]. The Ebsteinoid variant is considerably less common and is characterized by dysplastic leaflet morphology without the typical apical displacement required for the diagnosis of classical Ebstein anomaly [5]. Recognition of this distinction is important because surgical repair should be individualized according to valve morphology.

Our patient presented with an exceptionally rare combination of an Ebsteinoid tricuspid valve anomaly, giant right atrial aneurysm, and secundum ASD. Although right atrial enlargement frequently accompanies severe tricuspid regurgitation, aneurysmal enlargement of this magnitude has only rarely been described. To our knowledge, only a few cases of anterior displacement of the tricuspid valve anterior leaflet or displacement of all three tricuspid valve leaflets have been described in the literature (5,6). Chronic right-sided volume overload due to severe tricuspid regurgitation, compounded by left-to-right shunting across the ASD, likely contributed to progressive aneurysmal dilatation. Multimodality imaging was essential for diagnosis. Transthoracic echocardiography established the presence of severe tricuspid regurgitation and abnormal valve morphology, whereas four-dimensional echocardiography provided superior assessment of leaflet motion and coaptation. Cardiac CT accurately delineated the extent of aneurysmal enlargement and excluded associated vascular abnormalities, facilitating surgical planning [3, 7].

### Hemodynamic discrepancy and echocardiographic considerations

A pivotal observation in this case was the marked discrepancy between the echocardiographic estimation of right ventricular systolic pressure (RVSP) and the invasive catheterization findings. On continuous-wave Doppler, a peak regurgitant velocity of 3.39 m/s yielded a simplified Bernoulli transvalvular gradient of 46 mmHg, which led to an initial echocardiographic impression of moderate pulmonary hypertension. However, right heart catheterization conclusively revealed normal right ventricular and pulmonary artery pressures (32/6 mmHg and 28/12 mmHg, mean 18 mmHg, respectively), with low pulmonary vascular resistance (1.1 Wood units·m²).

This discordance underscores well-recognized limitations of Doppler-derived pressure calculations in complex tricuspid pathology:

#### Jet Eccentricity and Wall Impingement

In severe low-pressure TR, eccentric jets striking the compliant atrial wall or leaflet edges can produce localized velocity spikes that overestimate true transvalvular pressure gradients.

#### Gradient Versus Absolute RVSP

The Doppler value of 46 mmHg represented the transvalvular systolic pressure gradient alone rather than an additive RVSP calculation; factoring in an assumed elevated right atrial pressure further compounded the overestimation of pulmonary pressures.

#### Giant Atrial Compliance

The massive, highly compliant right atrial aneurysm effectively buffered regurgitant volume at low filling pressures (mean RA pressure 8 mmHg on catheterization), markedly altering regurgitant velocity kinetics.

Cardiac catheterization remains essential when noninvasive estimations present conflicting assessments of pulmonary vascular resistance prior to definitive surgical repair.

The principal goals of surgery were restoration of tricuspid valve competence, elimination of intracardiac shunting, and reduction of the massively dilated right atrium. Contemporary management of Ebstein anomaly increasingly favors valve repair over replacement because preservation of the native valve provides superior long-term right ventricular function and avoids prosthesis-related complications, particularly in young patients [2, 8]. Right atrial reduction atrioplasty is an effective adjunctive procedure that decreases atrial volume, reduces wall stress, improves cardiac geometry, and may reduce the future risk of atrial arrhythmias [6].

Early surgical intervention before irreversible right ventricular dysfunction develops is associated with excellent functional recovery and improved long-term outcomes [2, 8]. Our patient demonstrated marked symptomatic improvement, stable valve function, and preserved right ventricular performance one year after surgery.

This case emphasizes the importance of multidisciplinary management involving pediatric cardiologists, cardiac surgeons, imaging specialists, and anesthesiologists. Individualized surgical planning based on comprehensive anatomical assessment resulted in an excellent early outcome despite the complexity of the congenital abnormalities.

The principal limitation of this report is the relatively short duration of follow-up. Continued surveillance is required to evaluate long-term durability of valve repair, right ventricular function, and the occurrence of late arrhythmias.

## Conclusion

Giant right atrial aneurysm secondary to an Ebsteinoid tricuspid valve anomaly associated with a secundum ASD is an exceptionally rare congenital cardiac lesion. Comprehensive multimodality imaging accurately defined the complex anatomy and guided successful surgical repair. Combined tricuspid valve repair, ASD closure, and right atrial reduction atrioplasty resulted in excellent clinical and echocardiographic outcomes. Early recognition and timely intervention are essential to prevent progressive right-sided heart failure and arrhythmic complications.

## Learning objectives

1. **Morphological Differentiation**: Differentiate an Ebsteinoid tricuspid valve anomaly (abnormal leaflet morphology without classical apical displacement or ventricular atrialization) from typical Ebstein anomaly.
2. **Diagnostic Evaluation**: Explain the role of multimodality imaging (4D echocardiography, cardiac CT) and cardiac catheterization in assessing anatomical details, pulmonary hemodynamics, and surgical operability.
3. **Surgical Management & Outcomes**: Identify the key surgical procedures (tricuspid valve repair, ASD closure, and right atrial reduction atrioplasty) required to achieve long-term functional recovery and symptom relief.

## Supplementary Information

Below is the link to the electronic supplementary material.

Supplementary Material 1

Supplementary Material 2

## Data Availability

No datasets were generated or analysed during the current study.

## Abbreviations

RAA: Right atrial aneurysm
ASD: Atrial septal defect
NYHA: New York Heart Association
ECG: Electrocardiography
2D ECHO: Two dimensional Echocardiography
CT: Computed tomography
MRI: Magnetic resonance imaging (MRI)
